# Rapid and dynamic detection of endogenous proteins through *in locus* tagging in rice

**DOI:** 10.1016/j.xplc.2024.101040

**Published:** 2024-07-20

**Authors:** Yifu Tian, Dating Zhong, Rundong Shen, Xinhang Tan, Chen Zhu, Kai Li, Qi Yao, Xinbo Li, Xuening Zhang, Xuesong Cao, Pengcheng Wang, Jian-Kang Zhu, Yuming Lu

**Affiliations:** 1 Shanghai Collaborative Innovation Center of Agri-Seeds, Joint Center for Single Cell Biology, School of Agriculture and Biology, Shanghai Jiao Tong University, Shanghai 200240, China; 2 Ministry of Agriculture and Rural Affairs Key Laboratory of Gene Editing Technologies (Hainan), Institute of Crop Sciences and National Nanfan Research Institute, Chinese Academy of Agricultural Sciences, Sanya, Hainan 572024, China; 3 Shanghai Center for Plant Stress Biology, CAS Center for Excellence in Molecular Plant Sciences, Chinese Academy of Sciences, Shanghai 201602, China; 4 Institute of Advanced Biotechnology and School of Medicine, Southern University of Science and Technology, Shenzhen 518055, China; 5 Hainan Seed Industry Laboratory, Sanya, Hainan 572024, China

## Abstract

Understanding the behavior of endogenous proteins is crucial for functional genomics, yet their dynamic characterization in plants presents substantial challenges. Whereas mammalian studies have leveraged *in locus* tagging with the luminescent HiBiT peptide and genome editing for rapid quantification of native proteins, this approach remains unexplored in plants. Here, we introduce the *in locus* HiBiT tagging of rice proteins and demonstrate its feasibility in plants. We found that although traditional HiBiT blotting works in rice, it failed to detect two of the three tagged proteins, a result attributable to low luminescence activity in plants. To overcome this limitation, we engaged in extensive optimization, culminating in a new luciferin substrate coupled with a refined reaction protocol that enhanced luminescence up to 6.9 fold. This innovation led to the development of TagBIT (tagging with HiBiT), a robust method for high-sensitivity protein characterization in plants. Our application of TagBIT to seven rice genes illustrates its versatility on endogenous proteins, enabling antibody-free protein blotting, real-time protein quantification via luminescence, *in situ* visualization using a cross-breeding strategy, and effective immunoprecipitation for analysis of protein interactions. The heritable nature of this system, confirmed across T_1_ to T_3_ generations, positions TagBIT as a powerful tool for protein study in plant biology.

## Teaser

This study introduces TagBIT, a method for *in locus* HiBiT tagging of endogenous rice proteins for the rapid and dynamic characterization via luminescence detection. TagBIT is heritable across generations and enables antibody-free blotting, real-time quantification, *in situ* visualization, and immunoprecipitation, making it a versatile tool for plant biology research.

## Introduction

Protein abundance and protein–protein interactions (PPIs) change dynamically during cellular signaling, directly affecting biological processes (Li et al., 2018; Jiang et al., 2019). However, the scarcity of reliable antibodies and the lack of visualization technologies hinder the characterization of endogenous proteins (Lu et al., 2020a; Savic et al., 2015). Transgenic strategies are commonly used to express epitope-tagged proteins in plants. This has greatly facilitated the study of proteins. However, despite being driven by an endogenous promoter, transgene expression may not fully recapitulate the endogenous characteristics of the protein of interest (Butel et al., 2021; Feng et al., 2021).

The advent of precise editing technologies (Kumar et al., 2023; Li et al., 2022, Li et al., 2023; Lu et al., 2020b; Tian et al., 2023; Zong Y., Liu Y., Xue C., Li B., Li X., Wang Y., Li J., Liu G., Huang X., Cao X., 2022) has led to CRISPR‒Cas9-based *in locus* tagging, enabling researchers to unravel the functions of endogenous proteins (Artegiani et al., 2020; Bukhari and Müller, 2019; Sarov et al., 2012). Delivery of chemically modified donor DNA through bombardment, or the use of *Agrobacterium* to deliver optimized prime editing systems, enables seamless *in locus* tagging of endogenous proteins with Flag or HA tags, significantly reducing the difficulty of studying endogenous proteins in plants (Kumar et al., 2023; Li et al., 2023; Lu et al., 2020b; Tian et al., 2023; Zong Y., Liu Y., Xue C., Li B., Li X., Wang Y., Li J., Liu G., Huang X., Cao X., 2022).

**Figure 1:**
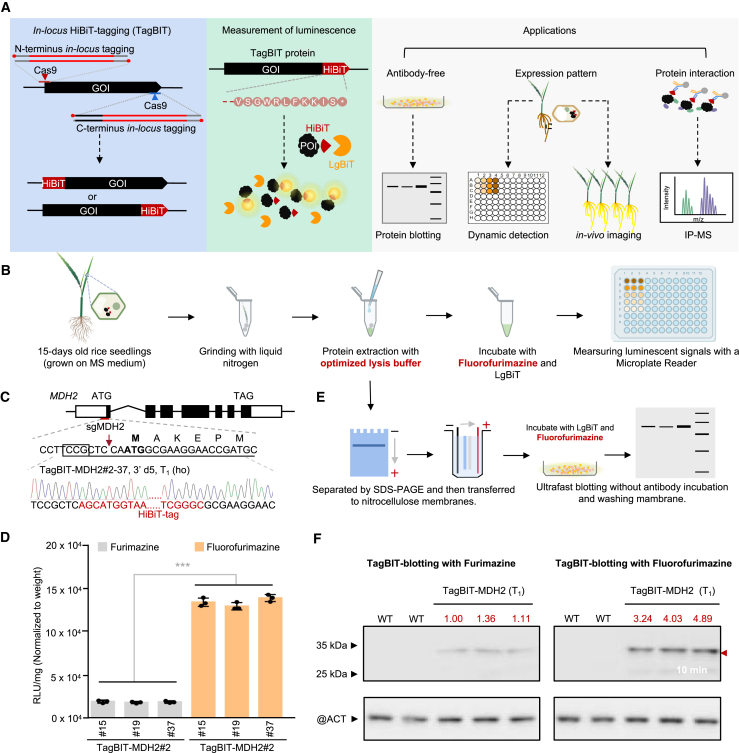
Optimization of the TagBIT system for enzymatic quantification of endogenous protein abundance **(A)** Overview of the TagBIT system and its applications in plants.**(B)** Schematics of the workflow for the TagBIT-based *in vitro* assay.**(C)***In locus* tagging of MDH2 with HiBiT in rice. The T_1_ homozygous line (TagBIT-MDH2 #2–37) was genotyped by Sanger sequencing. The sgRNA target is underlined, and the PAM is marked in the box. The start codon and first amino acid residue are marked in bold. 3′ d5 indicates a 5-nucleotide deletion at the 3′-junction (the same below).**(D)** Optimization of the TagBIT-based enzymatic quantification system. T_1_ homozygous lines were used as the tested samples. Values indicate means ± SD (*n* = 3). Statistical analyses were performed using Student’s *t*-test (∗∗∗*p* < 0.001).**(E)** Schematics of the workflow for TagBIT-based protein blotting.**(F)** Protein blotting of MDH2 with the optimized TagBIT system. Compared with the blotting result (left) using the traditional luminescence substrate (Furimazine) and buffers, the blotting signal was increased when an optimized substrate (Fluorofurimazine) and buffers were used (right). The number above each band represents the relative signal density of MDH2 normalized to OsActin (below) calculated with ImageJ, with the blotting signal with Furimazine counted as 1.

General epitope tags such as Flag and HA are widely used in immunoaffinity-based protein detection, isolation, and purification because of their high affinity and specificity with corresponding antibodies (Vandemoortele et al., 2019). However, antibody-dependent immunoblotting and immunohistochemistry require intricate procedures that limit the rapid and dynamic detection of endogenous proteins *in vitro* and *in vivo*. In this study, we developed a method, termed TagBIT, for the quantitative and dynamic analysis of endogenous rice proteins through *in locus* tagging with a luciferase-derived 11-amino-acid peptide (VSGWRLFKKIS, HiBiT tag; Schwinn et al., 2018) (Figure 1A). HiBiT-tagged proteins can be easily and sensitively detected *in vitro* and *in vivo* through the spontaneous complementation of HiBiT and LgBiT to restore luciferase activity (Schwinn et al., 2018). We optimized the procedures for TagBIT-based antibody-free protein blotting, dynamic quantification of protein abundance, and *in situ* bioluminescence visualization, thus considerably facilitating protein quantification in rice. In addition, TagBIT-based immunoprecipitation–mass spectrometry (IP–MS) extends the application of TagBIT for endogenous PPI analysis (Figure 1A).

## Results

### Development of the TagBIT system in plants

The small size of HiBiT and its high affinity to LgBiT render it an ideal tag for *in locus* tagging through CRISPR‒Cas9, ensuring the minimal perturbation of endogenous proteins and avoiding the introduction of potential overexpression artifacts (Lu et al., 2020a; Savic et al., 2015). Recently, several CRISPR-based targeted insertion methods have reduced the difficulty of *in locus* tagging, making it a common technique for the study of plant endogenous proteins (Kumar et al., 2023; Li et al., 2023; Tian et al., 2023). As reported previously (Lu et al., 2020b), chemically modified dsODNs (double-strand oligodeoxynucleotides) significantly improve targeted insertion efficiency, thus facilitating *in locus* tagging in rice (Figure 1A).

We designed a universal 57-bp dsODN, chemically modified to enhance integration efficiency, for N-terminal HiBiT tagging of three rice proteins: MDH2, TT1, and SLR1 (Supplemental Figure 1). Following our previously established methodology, we co-delivered 0.5 pmol of the modified donor with corresponding CRISPR‒Cas9 plasmids into rice calli through particle bombardment. Subsequent regeneration yielded 222 T_0_ plantlets, which were individually subjected to PCR analysis. A subset of 49 plantlets exhibiting larger PCR products were subjected to Sanger sequencing, which confirmed that 13 of the plantlets (5.9%) harbored the desired TagBIT insertions (Supplementary Table 1).

To evaluate the quantitative performance of the TagBIT system and its potential for use in rapid protein blotting assays, we focused on screening for T_1_ homozygotes of the tagged genes (Supplementary Table 2). In initial trials using a commercial detection kit for HiBiT-tagged proteins, we encountered problems of low signal intensities and limited stability, which posed challenges for the quantification of low-abundance proteins such as TT1 and SLR1 (Supplemental Figures 2A–2C). The HiBiT blotting technology, favored for its antibody-free detection and simplification of the traditionally laborious western blotting method (Ozono et al., 2020), was also found to be insufficient for detection of low-expressed proteins in our initial attempts with the commercial method (Supplemental Figure 2D).

**Figure 2:**
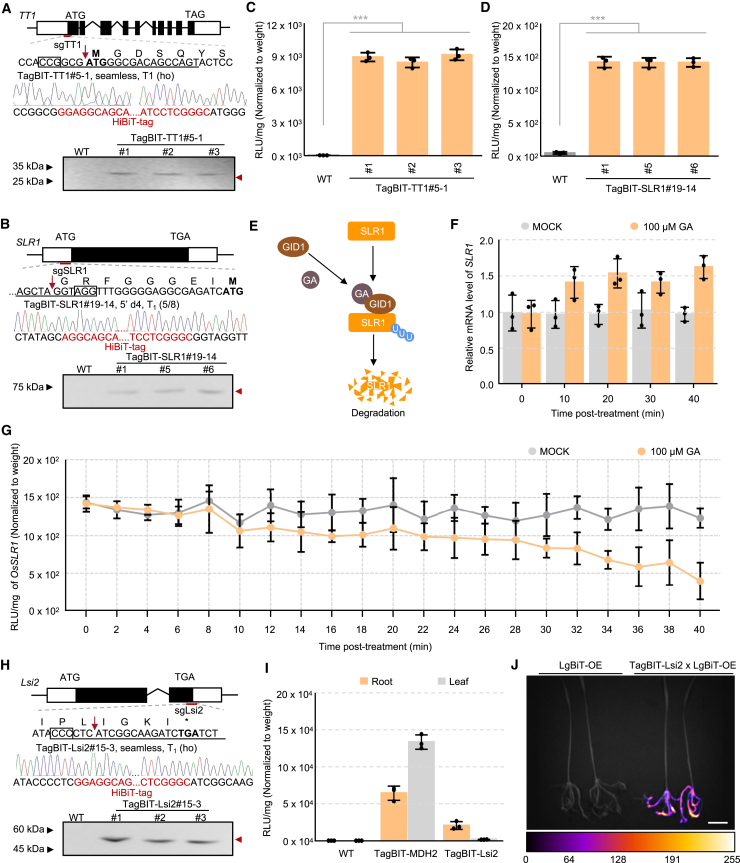
TagBIT-based dynamic quantification and *in situ* imaging of endogenous proteins **(A** and **B)** Schematic diagram of TagBIT of TT1 **(A)** and SLR1 **(B)**. Protein blotting results of the proteins (shown in lower panel).**(C** and **D)** TagBIT-based abundance quantification of TagBIT-TT1 **(C)** and TagBIT-SLR1 **(D)**. Values indicate means ± SD (*n* = 3). Statistical analyses were performed using Student’s *t*-test (∗∗∗*p* < 0.001).**(E)** Schematic diagram showing the GA-dependent degradation of SLR1.**(F** and **G)** Dynamic quantification of *SLR1* transcript **(F)** and protein **(G)** abundance in transgene-free T_3_ homozygotes with or without GA treatment. The *ACTIN1* gene (Os03g0718100) was used as an internal control in qPCR. Values indicate means ± SD (*n* = 3).**(H)** TagBIT of the *Lsi2* gene in rice. The T_1_ homozygous mutant Lsi2#15-3 was identified by Sanger sequencing. TagBIT-based protein blotting of Lsi2 in T_2_ homozygotes is shown below.**(I)** Quantification of TagBIT protein abundance in root and leaf tissue. T_1_ homozygous mutants were used, and values indicate means ± SD (*n* = 3).**(J)** Bioluminescence image of Lsi2 protein in hybrid F_1_ seedlings (TagBIT-Lsi2 × LgBiT-OE). The color bar indicates the arbitrary luminescence intensity corresponding to each pseudo color. Scale bar, 3 cm.

To address these issues, the TagBIT-MDH2 plants were subjected to a refined TagBIT system to increase detection efficiency (Figures 1B and 1C; Supplemental Figure 2E). We posited that the strong pectin-rich cell walls characteristic of plant tissues necessitate more effective lysis techniques than those typically used for mammalian samples. Effects of interfering substances such as polyphenols, reactive oxygen species, and phytochromes known to affect enzymatic reactions (Imlay, 2013; Hügel et al., 2016; Khakhar et al., 2020) were mitigated by thorough tissue grinding in liquid nitrogen and use of a bespoke lysis buffer tailored for plant samples (see methods). Our optimization efforts were further improved by the discovery that Fluorofurimazine (Gaspar et al., 2021; Su et al., 2023), a variant of the commercial Furimazine substrate, significantly boosts performance in plant assays. Through these enhancements, TagBIT detection efficiency was notably improved, showing a 6.9-fold increase (Figure 1D). Moreover, antibody-free protein blotting with the optimized system revealed an enhanced signal for MDH2 (Figures 1E and 1F). Antibody-based western blotting validated the TagBiT blotting results (Supplemental Figure 3). In addition, TT1 and SLR1 proteins, previously undetectable with traditional commercial kits in TagBiT blotting (Supplemental Figure 2D), were successfully identified using our improved TagBIT system, confirming its effectiveness for both enzymatic detection and blotting analysis of plant proteins (Figure 2A–2D).

Further validation of the TagBIT system was conducted on two additional rice genes with low expression, *BZR1* and *Nramp5* (Supplemental Figure 4A and 4B). Using the same gene-editing strategy, we obtained TagBIT plants for these genes. The progenies from T_1_ homozygotes (BZR1#7-5, Nramp5#10-7) were used for experimental assays (Supplemental Figure 4C–4F). The results were clear: both blotting and enzymatic detection by our TagBIT system detected these proteins, underscoring the broad applicability of our system for plant protein research.

### Dynamic tracking of endogenous proteins

As shown in Figure 2D, we successfully employed TagBIT technology to quantify the abundance of the target protein SLR1, a pivotal component of the gibberellin (GA) signaling pathway. GA is known to induce rapid degradation of SLR1 (Figure 2E; Hirano et al., 2008). However, tracking dynamic changes in the abundance of such post-translationally regulated proteins presents a significant challenge. Our TagBIT approach, which enables rapid quantification of protein levels via enzymatic detection, has made dynamic tracking of plant proteins feasible.

To validate the functionality of this system, we performed a real-time analysis of TagBIT-tagged SLR1 protein. T_2_ homozygous seedlings were treated with gibberellin GA_3_, and their response was monitored. Whereas qPCR analysis revealed that GA_3_ treatment enhanced SLR1 transcript levels by 1.5 fold (Figure 2F), TagBIT-mediated enzymatic assays showed a contrasting result: protein levels decreased rapidly within 40 min, undergoing a reduction of 61.6% (Figure 2G). This observation confirmed the presence of GA-dependent SLR1 protein degradation. These results highlight a critical insight—that mRNA abundance does not necessarily correlate with protein abundance, particularly in cases where post-translational regulation plays a significant role (Buccitelli and Selbach, 2020; Chen et al., 2021; Fortelny et al., 2017). These results suggest that TagBIT technology provides a rapid and straightforward solution for real-time tracking of protein abundance in plants.

### *In situ* visualization of target proteins

The study of endogenous protein distribution within its native cellular context is critical for understanding protein function. Traditional immunochemical staining techniques, reliant on specific antibodies for spatial detection, often pose challenges in plants. The HiBiT tag, with its luminescence properties, has shown promise for *in situ* protein visualization in mammalian studies, necessitating the presence of the membrane-impermeable LgBiT within the cell (White et al., 2020).

We postulated that the creation of transgenic plants expressing LgBiT could facilitate *in situ* protein detection when they were crossed with TagBIT plants. We therefore generated transgenic lines constitutively expressing LgBiT (LgBiT-OE) and obtained stable T_1_ progeny. To test this method, we focused on *Lsi2*, a gene with root-specific expression in rice (Tang et al., 2023). A rice line was engineered with a C-terminal TagBIT fusion to Lsi2 (Figures 2H and 2I). The homozygous lines were crossed with LgBiT-OE, and the F_1_ seedlings (TagBIT-Lsi2 × LgBiT-OE) were subjected to *in situ* bioluminescence assays (Supplemental Figure 5). Immersing the F_1_ seedlings in a Fluorofurimazine solution and imaging with a cryogenic CCD camera revealed specific localization of Lsi2 in the roots (Figure 2J), confirming the efficacy of our approach. This universal cross-breeding strategy enables effective *in situ* visualization of plant proteins and offers a versatile tool for characterizing the distribution of endogenous proteins in plants.

### Analyses of endogenous protein interactions

To investigate the utility of TagBIT technology for detection of endogenous PPIs in plants, we examined its potential for use in IP–MS assays. Although HiBiT and LgBiT exhibit a strong interaction within the TagBIT system, we found that LgBiT alone did not effectively pull down target proteins, suggesting that additional interaction strength is required for this application. Consequently, we explored the use of an anti-HiBiT antibody for IP–MS experiments to detect endogenous TagBIT proteins and their interaction partners.

**Figure 3:**
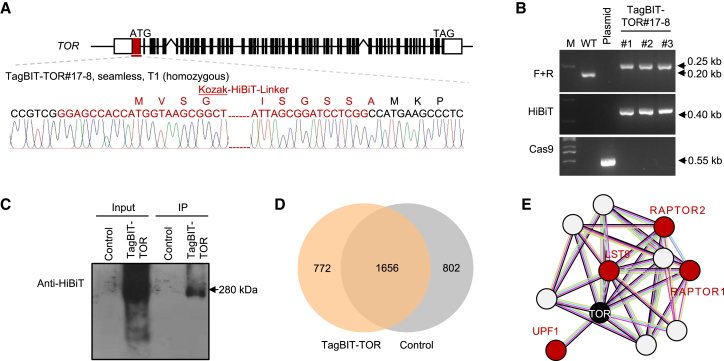
TagBIT-based IP–MS of OsTOR **(A)** TagBIT of the *TOR* gene in rice. The T_1_ homozygous mutant TOR#17-8 was identified by Sanger sequencing.**(B)** Screening of Cas9-free TagBIT homozygotes in T_2_ seedlings for IP–MS. Primers amplifying the flanking genomic sequence (F + R, upper), HiBiT (middle), and Cas9 (lower) were used for genotyping.**(C)** Protein blotting of TOR in an immunoprecipitation assay using HiBiT antibody. Leaves collected from 1-month-old T_3_ plants were used for protein extraction.**(D)** Venn diagram of TOR-interacting proteins identified by IP–MS.**(E)** Potential networks of TOR-interacting proteins revealed by the PPI STRING database; proteins detected by TagBIT-based IP–MS are marked in red.

We chose the *Target of Rapamycin* (*TOR*) gene, which plays fundamental roles in energy metabolism, crop yield, disease resistance, and stress tolerance (Li et al., 2024; Liu and Sabatini, 2020) and is particularly challenging to study because of its large size (∼26 kb). Using gene-editing techniques, we successfully generated edited plants with an N-terminal TagBIT-tagged TOR and isolated transgene-free T_2_ homozygotes (Figure 3A and 3B). We then performed IP–MS experiments on T_3_ seedlings using the HiBiT antibody (Figure 3C). Analysis of the IP products by mass spectrometry revealed a network of 772 potential interacting proteins (Figure 3D), confirming the effectiveness of the TagBIT system. Noteworthy among these proteins were established TOR interactors such as RAPTOR1, RAPTOR2, LST8, and UPF1 (Figure 3E). This study provides the first insights into the PPI landscape of the endogenous TOR protein in rice, indicating that TagBIT technology is a viable approach for identifying interactions among endogenous proteins, even those encoded by large genes.

## Discussion

Advances in nucleic-acid-based research techniques such as qPCR and high-throughput sequencing (NGS) technologies have significantly propelled genomics studies. In the post-genomics era, the focus has increasingly shifted toward proteins. We successfully developed the TagBIT technique for the study of endogenous proteins in plants, addressing the growing demand for robust and sensitive protein research methodologies (Figure 1A).

By greatly enhancing the sensitivity of plant protein detection, TagBIT can facilitate ultra-rapid, highly sensitive, and quasi-real-time detection of a wide array of plant proteins in a manner akin to luciferase assays (Figure 1B). The development of a TagBIT-based plant protein blotting method, which obviates the need for antibodies and simplifies the traditional process of western blotting, has markedly reduced the time required for protein blotting (Figure 1E). The complementation of HiBiT with LgBiT is essential for NanoLuc signal generation. By contrast, the non-specific binding of LgBiT does not produce a signal, resulting in a theoretically higher signal-to-noise ratio compared with that of antibody-based western blotting. Furthermore, our construction of LgBiT-OE lines enabled a TagBIT × LgBiT hybridization strategy for *in situ* visualization of the spatiotemporal distribution of endogenous plant proteins (Supplemental Figure 5). The application of a monoclonal antibody against HiBiT also facilitated the study of PPIs in plants using TagBIT technology.

The recent advent of GRAND editing, a technique based on prime editing for *Agrobacterium*-mediated targeted sequence knockin (Li et al., 2023; Liu et al., 2024; Zong Y., Liu Y., Xue C., Li B., Li X., Wang Y., Li J., Liu G., Huang X., Cao X., 2022), has opened new avenues for precision tagging of plant genes with HiBiT. This method is more cost-effective and results in lower copy number integration compared with particle bombardment. In addition, the unpredictable insertion orientations of donor fragments caused by particle bombardment may reduce TagBiT efficiency. Further optimization of *Agrobacterium*-mediated targeted insertion will provide a precise and high-throughput strategy for TagBiT in plants.

Because plant research necessitates stable heritability (Savic et al., 2015), the genetic stability observed from the T_1_ to T_3_ generations in our TagBIT plants is encouraging. Our results indicate that TagBIT is heritable without compromising the stability of protein detection across generations (Supplemental Figure 6). Although editing at untranslated regions can alter gene expression levels (Wang et al., 2024) and HiBiT knockin also affected the transcript levels of certain genes (e.g., TT1) in this study (Supplemental Figure 6C), our extensive qPCR analyses suggest that the overall impact on the transcript levels of tagged genes is minimal.

In summary, the TagBIT technology developed in this study is capable of addressing the complexities associated with various types of research on endogenous plant proteins. The speed, simplicity, and stability of the technology make it a potentially routine method in future plant research endeavors. The potential of TagBIT technology to streamline the study of plant proteins, combined with continued advances in plant gene-editing techniques, heralds a new era of precision protein research in plant science.

## Methods

### Plasmid construction

The pCBSG032 vector was used for targeted knockin and TR-HDR. The 23-bp targeting sequences (including the PAM) were selected within the target regions, and their targeting specificity was analyzed using CRISPR-P 2.0 (Liu et al., 2017). The sgRNA expression cassettes were constructed as described previously (Lu et al., 2020b). The dsODNs (Supplemental Table 3) used in this study were modified and synthesized by Sangon Biotech.

The LgBiT overexpression vector (pOE-LgBiT, Supplemental Sequence 1) was constructed from the pCambiaUBI-01 vector. The plant codon-optimized LgBiT sequence was synthesized (Genwiz), amplified, and used to replace SpCas9 by *Pst*I+*BamH*I digestion (NEB) and Gibson Assembly (Vazyme).

### Rice transformation and regeneration

For *Agrobacterium*-mediated transformation, *Agrobacterium tumefaciens* strain EHA105 was transformed with the relevant binary vectors (pUBI-LgBiT vector) using the freezing/heat shock method. *Agrobacterium*-mediated transformation of callus cells of the japonica rice variety Zhonghua 11 (ZH11) was performed as reported previously (Tian et al., 2023). Hygromycin B (50 mg/L; YEASEN, Shanghai, China) was used to select hygromycin-resistant calli. Plantlets were regenerated from hygromycin-resistant calli using the routine rice transformation method described previously (Tian et al., 2023). Biolistic transformation was performed using a PDS1000/He particle bombardment system (Bio-Rad) with a target distance of 6.0 cm from the stopping plate at a helium pressure of 7.5 MPa. 5′-Phosphorylated and phosphorothioate-modified oligos were synthesized (Sangon Biotech) and prepared as described previously (Lu et al., 2020b). To reduce random insertions, 0.5 pmol dsODN for each target was used for targeted knockin.

### Genotyping

Genomic DNA was extracted using the CTAB method. Leaf tissues were harvested from three different tillers of each plant (∼2 mg from each tiller) and mixed for DNA extraction. The primer pair KIF+KIR (Supplementary Table 3) flanking the target site was used for detection of targeted insertion events; KIF+KIR PCR products with the desired molecular weight were gel-purified (Vazyme) and cloned into the TA cloning vector pEasy (TransGen Biotech, Beijing, China). To verify the *in locus* tagging events, eight positive colonies for each selected plant sample were individually Sanger sequenced. The primers F2+R2 (Supplementary Table 3) were used for detection of editing efficiencies, and the mutations were discriminated using DSDecode (Liu et al., 2015). The PCR products were subjected to Sanger sequencing directly.

### *In vitro* lysis assay

The *in vitro* lysis assay for plants was modified from the Nano-Glo HiBiT Lytic Detection System (Promega, N3030). For rapid detection of the TagBIT proteins in rice plants, tissues of 15-day-old seedlings were collected and ground in liquid nitrogen. For stable and reproducible measurements, powdered plant tissue (∼20 mg) was mixed with optimized lysis buffer at a ratio of 1 mg/10 μl. The optimized lysis buffer was made by mixing equal volumes of Nano-Glo HiBiT Lytic Buffer (Promega, N247A) and dilution buffer (10 mM PBS buffer with 0.1% PVP-40 [pH 7.4]; Supplemental Figure 2E). After centrifugation at 12 000 *g* for 5 min, 100 μL of supernatant was collected and mixed with 1 μL LgBiT protein (Promega, N401A) and 2 μL Nano-Glo HiBiT Lytic Substrate (Promega, N246A). In the optimized procedure, Fluorofurimazine (MedChemExpress, HY-D1282) was formulated as a clear solution (5 mM stock) in 10% ethanol, 10% glycerol, 10% hydroxypropyl-β-cyclodextrin (Sigma-Aldrich, H5784), 35% PEG-300, and 35% water. Plant tissue lysate supernatant (100 μL) was mixed with 1 μL LgBiT protein (Promega, N401A) and 2 μL Fluorofurimazine solutions. After waiting at least 10 min for equilibration of LgBiT and HiBiT, the luminescence of the mixture was measured on a GloMAX 96 Microplate Luminometer (Promega). The relative luminescence unit (RLU) indicates the raw luminescence signal minus the assay background (blank) and is normalized to tissue weight (RLU/mg). For dynamic detection of TagBIT-SLR1, the 15-day-old transgene-free T_3_ rice homozygotes were divided into two equal aliquots; one received a foliar spray of 100 μM GA_3_ (Solarbio, G8040), and the other was sprayed with distilled water as a mock control.

### Protein extraction and protein blotting

To extract total protein from rice plants, 50 mg plant tissue was ground in liquid nitrogen and mixed with 200 μL plant lysis buffer (50 mM Tris–HCl [pH 7.5], 150 mM NaCl, 0.5 mM EDTA [pH 8.0], 10% glycerol, 0.5% Triton X-100, 10 mM PMSF, and 1% protease inhibitor cocktail). Extractions were clarified by centrifugation at 4°C for 10 min. The supernatant was mixed with an equal volume of 2× loading buffer, and the samples were boiled at 100°C for 5 min. For low-abundance proteins, i.e., SLR1, the protein solution could be concentrated with a concentrator (Eppendorf).

TagBIT-based protein blotting was modified from the Nano-Glo HiBiT blotting system. The protein lysates were separated by SDS–PAGE and transferred to nitrocellulose membranes. After the membranes were incubated with incubation buffer (10 mM TBS buffer [pH 7.4], 0.1% Tween-20, and 1 mM DTT) for 30 min, they were transferred into blotting buffer (Nano-Glo HiBiT blotting buffer; 1:200 LgBiT) and incubated for 1 h. Finally, Nano-Glo Luciferase Assay Substrate or Fluorofurimazine solution (5 mM stock) was diluted 500-fold into the blotting buffer, and the blot was imaged with a cooled CCD camera.

Western blotting was performed as described previously (Tian et al., 2023). OsMDH2 and OsActin proteins were immunoblotted with anti-OsMDH2 (Os10g0478200 antibody, PHY4019S; PhytoAB, San Jose, CA, USA) and anti-Actin (plant-specific, D191048; Sangon Biotech, Shanghai, China) antibodies.

### *In vivo* bioluminescence imaging

Fluorofurimazine was formulated as a clear solution in 10% ethanol, 10% glycerol, 10% hydroxypropyl-β-cyclodextrin (Sigma-Aldrich), 35% PEG-300, and 35% water. Seedlings co-expressing LgBiT and TagBIT were submerged in a 100 μM Fluorofurimazine solution at room temperature and then immediately imaged with a nitrogen-cooled CCD camera (Lumazone Pylon 2048B).

### TagBIT-based IP–MS

TagBIT-TOR T_3_ lines were used for the IP–MS assay. Leaf tissue (5 g) was harvested from 1-month-old rice seedlings, and total proteins were extracted with plant lysis buffer (50 mM Tris–HCl [pH 7.5], 150 mM NaCl, 0.5 mM EDTA [pH 8.0], 10% glycerol, 0.5% Triton X-100, and 10 mM PMSF). Extractions were then clarified by centrifugation at 4°C for 10 min. For IP, 20 μL Protein-A/G mag beads (MedChemExpress; HY-K0202) were incubated with 2 μL anti-HiBiT antibody (Promega, N7200) for 2–3 h with gentle rotation at 4°C; extractions of equal total proteins were then added, and the beads were incubated for an additional 3 h at 4°C. The beads were washed four times with wash buffer (50 mM Tris–HCl [pH 7.5], 100 mM NaCl, 0.5 mM EDTA [pH 8.0], and 0.1% Triton X-100). The protein samples were sent to the Core Facility for Proteomics at the Shanghai Center for Plant Stress Biology (PSC) for affinity purification and mass spectrometry as described previously (Wang et al., 2023) with two biological replicates. The putative interaction partners detected by TagBIT-IP/MS are listed in Supplementary Table 4.

### Statistical analysis

The relevant statistical tests, sample sizes, and replicate types for each analysis are found in the associated figure or table and/or the corresponding figure legends.

## Funding

This study was supported by the 10.13039/501100012166National Key R&D Program of China (no. 2021YFD1201300 to Y.L. and 2021YFA1300404 to J.-K.Z.), the 10.13039/501100001809National Natural Science Foundation of China (32070396 to Y.L.), and the 10.13039/501100002858China Postdoctoral Science Foundation (BX20220098 and 2022M720973 to Y.T.).

## Author contributions

Conceptualization, Y.T., Y.L., and J.-K.Z.; methodology, Y.T. and Y.L.; investigation, Y.T., D.Z., R.S., X.T., C.Z., K.L., Q.Y., X.L., X.Z., and X.C.; writing – original draft, Y.T. and Y.L.; writing – review & editing, Y.L. and J.-K.Z.; funding acquisition, Y.L., J.-K.Z., and Y.T.; resources, J.-K.Z. and P.W.; supervision, Y.L. and J.-K.Z.

## Supplemental information

Document S1. Figures S1–S6, Tables S1–S3, Supplementary Sequence S1, and Supplementary Files S1 and S2

Table S4. 772 putative interaction partners detected by TagBIT-IP/MS

Document S2. Article plus supplemental information
