# Supplementary material for: Rapid and dynamic detection of endogenous proteins through in locus tagging in rice
Source: Plant Commun. 2024 Jul 20;5(11):101040. doi: 10.1016/j.xplc.2024.101040 (PMC11589287; doi:10.1016/j.xplc.2024.101040)
Supplement: Document S1. Figures S1–S6, Tables S1–S3, Supplementary Sequence S1, and Supplementary Files S1 and S2 [file mmc1.pdf]

Plant Communications, Volume *5*

Supplemental information

Rapid and dynamic detection of endogenous proteins through *in locus* tagging in rice

Yifu Tian, Dating Zhong, Rundong Shen, Xinhang Tan, Chen Zhu, Kai Li, Qi Yao, Xinbo Li, Xuening Zhang, Xuesong Cao, Pengcheng Wang, Jian-Kang Zhu, and Yuming Lu

Supplementary figures and tables for

Rapid and dynamic detection of endogenous proteins through *in-locus* tagging in rice

Yifu Tian[1,2,5], Dating Zhong[1,3], Rundong Shen[2,5], Xinhang Tan[3], Chen Zhu[4], Kai Li[1], Qi Yao[1,3], Xinbo Li[2,5], Xuening Zhang[3], Xuesong Cao[4], Pengcheng Wang[4], Jian-Kang Zhu[2,4]* and Yuming Lu[1]*

[1]Shanghai Collaborative Innovation Center of Agri-Seeds, Joint Center for Single Cell Biology, School of Agriculture and Biology, Shanghai Jiao Tong University, Shanghai 200240, China.

[2]Ministry of Agriculture and Rural Affairs Key Laboratory of Gene Editing Technologies (Hainan), Institute of Crop Sciences and National Nanfan Research Institute, Chinese Academy of Agricultural Sciences, Sanya, Hainan 572024, China.

[3]Shanghai Center for Plant Stress Biology, CAS Center for Excellence in Molecular Plant Sciences, Chinese Academy of Sciences, Shanghai 201602, China.

[4]Institute of Advanced Biotechnology, and School of Life Sciences, Southern University of Science and Technology, Shenzhen 518055, China.

[5]Hainan Seed Industry Laboratory, Sanya, Hainan 572024, China.

*To whom correspondence should be addressed. E-mail: luymin@sjtu.edu.cn, zhujk@sustech.edu.cn.

Supplementary Materials

Supplementary Figure 1. DNA and amino acid sequences of representative TagBIT plants.

Supplementary Figure 2. Optimization of TagBIT-based lytic detection in rice.

Supplementary Figure 3. Comparison of TagBIT- and antibody-based protein blotting.

Supplementary Figure 4. Characterization of two additional endogenous proteins in rice using TagBIT.

Supplementary Figure 5. Schematics to show TagBIT-based in vivo bioluminescence imaging.

Supplementary Figure 6. Quantification of mRNA and protein level of representative TagBIT plants.

Supplementary Table 1. Summary of TagBIT efficiencies in T_0 plants.

Supplementary Table 2. Transmission frequencies of selected TagBIT lines.

Supplementary Table 3. The primers used in this study.

Supplementary Table 4. 772 putative interacting partners detected with TagBIT-IP/MS in this study.

Supplementary Sequence 1. DNA sequence of pOE-LgBiT vector.

Supplementary File 1. Unprocessed image of DNA electrophoresis.

Supplementary File 2. Unprocessed image of TagBIT blotting.

Supplementary figure 1. DNA and amino acid sequences of representative TagBIT plants.

Gene	Line	Sequence
OsMDH2	WT	......TTCCCCCTCTCTCGCAAAACTCACCCAAAAGAGCAGCGTCGCCTCTCCTCCTCCTCCCT<br>M A<br>AACCCCTACGCTTCCAGAACCTTCTCGAAGCTCCCGCTCCCCCCCCCCTTCCGCTCCAATGGCGA<br>K E P M R V L V T<br>AGGAACCGATGCGCGTGCTCGTCACC......
	TagBIT	......TTCCCCCTCTCTCGCAAAACTCACCCAAAAGAGCAGCGTCGCCTCTCCTCCTCCTCCCT<br>M V<br>AACCCCTACGCTTCCAGAACCTTCTCGAAGCTCCCGCTCCCCCCCCCCTTCCGCTCAGCATGGTA<br>S G W R L F K K I S G S S G A K E P M R V L<br>AGCGGCTGGCGGCTGTTCAAGAAGATTAGCGGATCCTCGGGCGCGAAGGAACCGATGCGCGTGCT<br>V T<br>CGTCACC......
OsTT1	WT	......GAGTCGGCGGCGACAACAAATCGAAAATCGAACGGGAGAAATTCTCTCTGATTCTCTCT<br>M G<br>CGCGGGCGGCGGAAGCAATCGTAGTTAGCAGAGGCCGGCGAGCGAGCCGCCACCGGCGATGGGCG<br>D S Q Y S F S L T<br>ACAGCCAGTACTCCTTCTCCCTCACC......
	TagBIT	......GAGTCGGCGGCGACAACAAATCGAAAATCGAACGGGAGAAATTCTCTCTGATTCTCTCT<br>CGCGGGCGGCGGAAGCAATCGTAGTTAGCAGAGGCCGGCGAGCGAGCCGCCACCGGCGGGAGGCA<br>M V S G W R L F K K I S G S S G M G D S Q<br>GCATGGTAAGCGGCTGGCGGCTGTTCAAGAAGATTAGCGGATCCTCGGGCATGGGCGACAGCCAG<br>Y S F S L T<br>TACTCCTTCTCCCTCACC......
OsSLR1	WT	......GCTTCCCAACCCTGGATCCAAATCCCAACCTATCCCAAAGCCGAAACCGAGGAGAGGAA<br>M K<br>AAAGGTTACGCGCAATTATTACTAGCTATAGCTAGGTAGGTTTGGGGGAGGCGAGATCATGAAGC<br>R E Y Q E A G G S<br>GCGAGTACCAAGAAGCCGGCGGGAGC......
	TagBIT	......GCTTCCCAACCCTGGATCCAAATCCCAACCTATCCCAAAGCCGAAACCGAGGAGAGGAA<br>M V S G W R L F K<br>AAAGGTTACGCGCAATTATTACTAGCTATAGCAGGCAGCATGGTAAGCGGCTGGCGGCTGTTCAA<br>K I S G S S G G R F G G G E I M K P S P H<br>GAAGATTAGCGGATCCTCGGGCGGTAGGTTTGGGGGAGGCGAGATCATGAAGCGCGAGTACCAAG<br>F P E I G<br>AAGCCGGCGGGAGC......
OsTOR	WT	......GCAAGCCAAGCCAGGTCGGGAAAGCTAAACCCTCCCCTCCCCCCTCCCTCTATCTCCGC<br>M K<br>CTCGGCCGCTCGCCGCCGCGTCGGCTCTGGAGCCGCCGCCGCCGCCGCCGCCGTCGCCATGAAGC<br>P S P H F P E I G<br>CCTCGCCGCACTTCCCGGAGATCGGG......
	TagBIT	......GCAAGCCAAGCCAGGTCGGGAAAGCTAAACCCTCCCCTCCCCCCTCCCTCTATCTCCGC<br>CTCGGCCGCTCGCCGCCGCGTCGGCTCTGGAGCCGCCGCCGCCGCCGCCGCCGTCGGGAGCCACC<br>M V S G W R L F K K I S G S S A M K P S P H<br>ATGGTAAGCGGCTGGCGGCTGTTCAAGAAGATTAGCGGATCCTCGGCCATGAAGCCCTCGCCGCA<br>F P E I G<br>CTTCCCGGAGATCGGG......

Supplementary Figure 1. Continued.

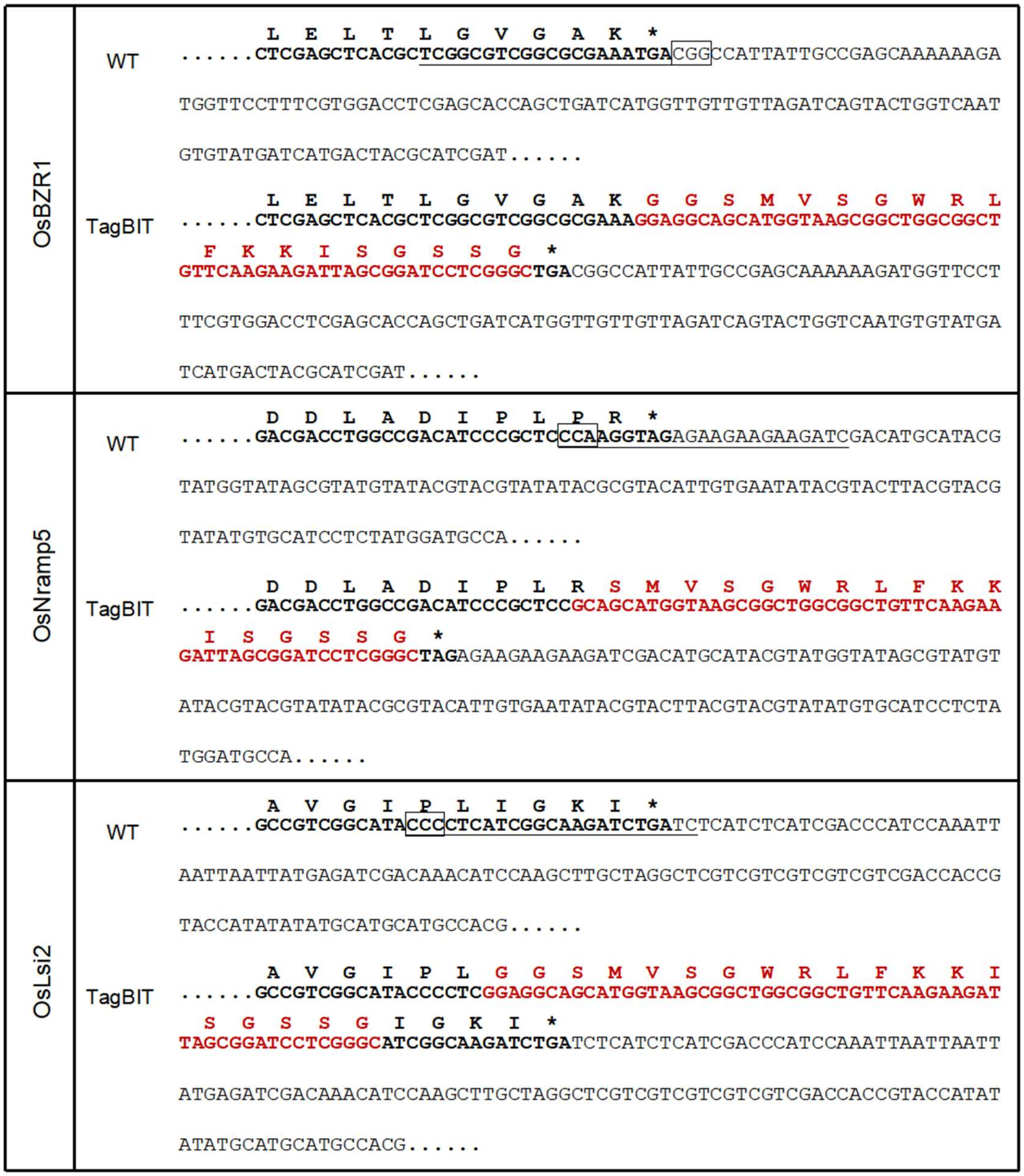

Note. Original DNA sequences and corresponding amino acid sequences were marked in bold. Inserted DNA sequences and corresponding amino acid sequences were marked in red. The sgRNA targets of Cas9 are underlined and the protospacer-adjacent motifs (PAMs) marked with boxes.

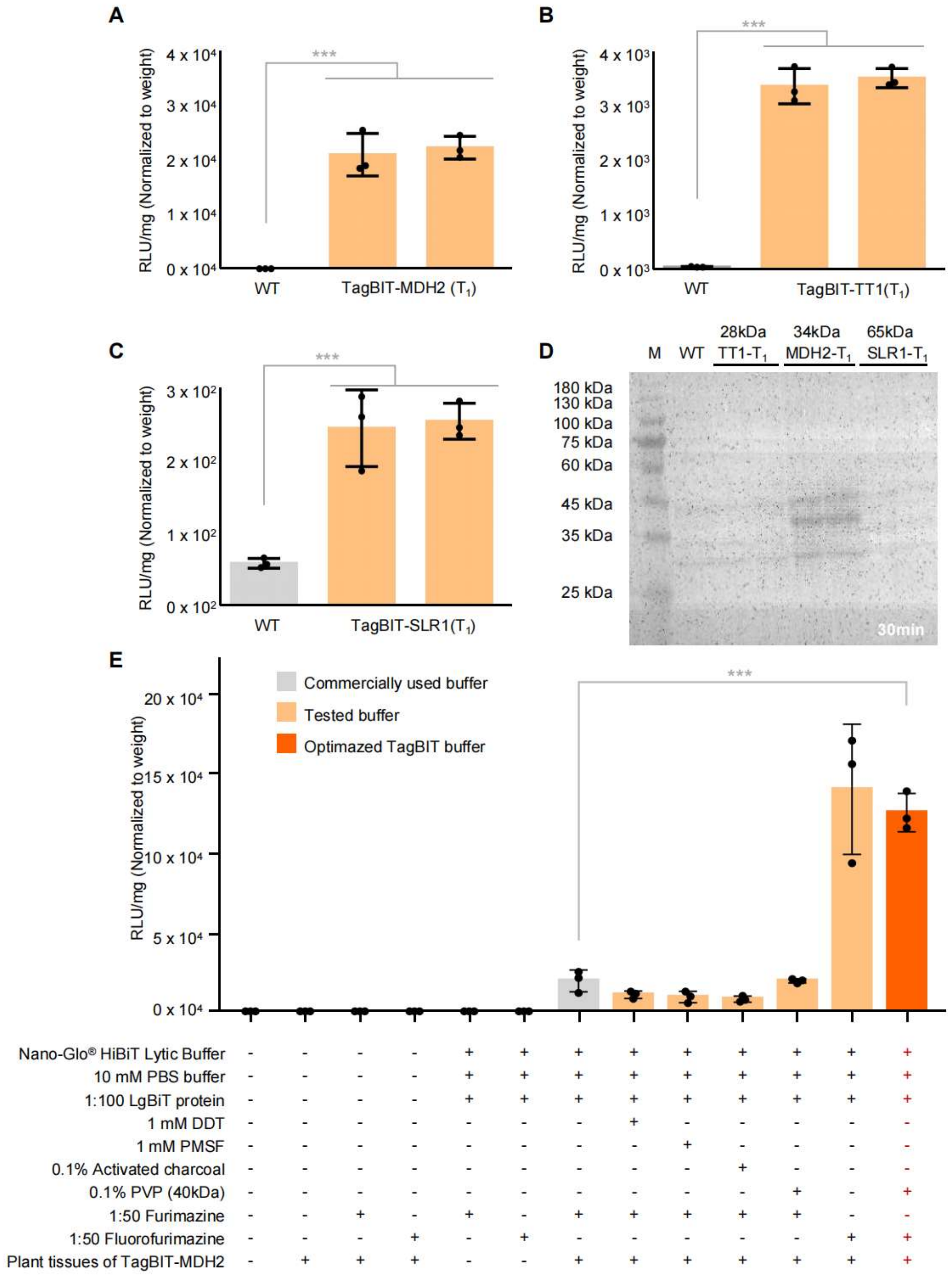

Supplementary Figure 2. Optimization of TagBIT-based lytic detection in rice. (A-C) HiBiT-based lytic detection of MDH2 (A), TT1 (B), and SLR1 (C) with commercial kit. **(D)** Protein blotting of three selected proteins with commercial HiBiT-blotting kit. **(E)** Comparison of *in vitro* lysis detection results with commercial and optimized lysis buffers. Values indicate means ± SD (n = 3). Statistical analyses were performed using Student's *t*-test: *** indicates $P < 0.001$.

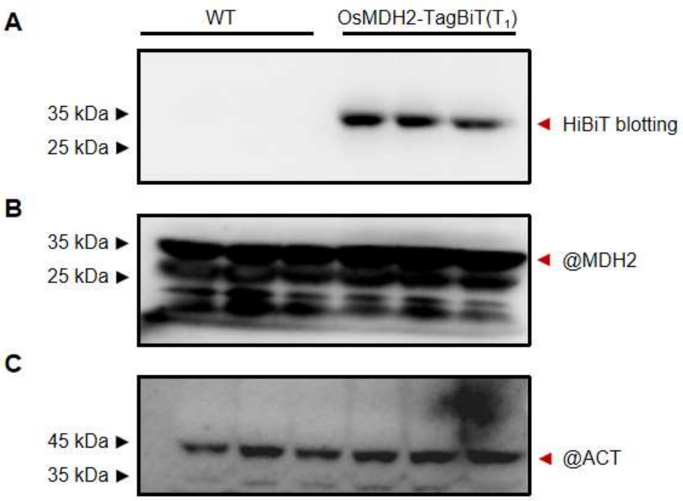

Supplementary Figure 3. Comparison of TagBIT- and antibody-based protein blotting. (A) HiBiT-based protein blotting for detection of TagBIT-MDH2. The optimized substrate (Fluorofurimazine) was used. **(B)** Western blotting of OsMDH2 with commercial antibody (Os10g0478200 antibody, PHY4019S; PhytoAB, San Jose, CA, USA). **(C)** OsActin was used as the loading control.

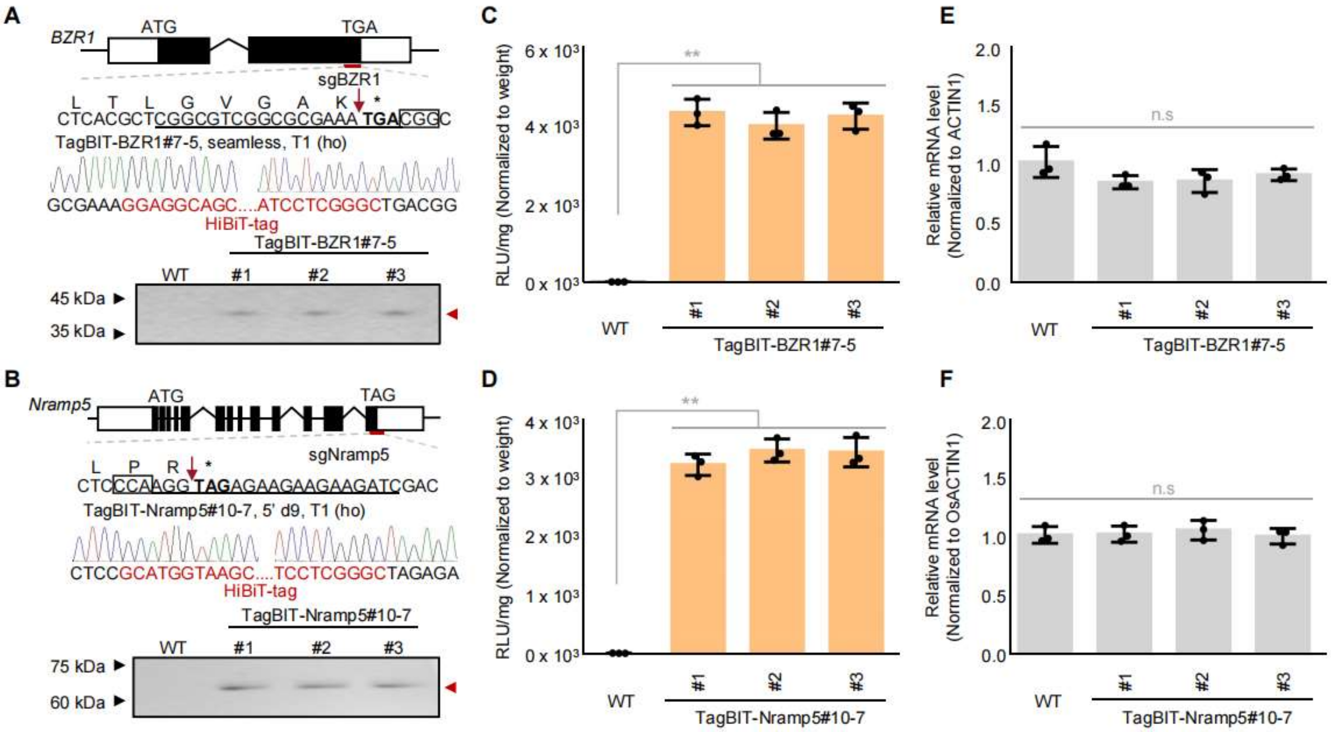

Supplementary Figure 4. Characterization of two additional endogenous proteins in rice using TagBIT. **(A-B)** Schematic diagram to show TagBIT of *BZR1* (A) and *Nramp5* (B). Protein blotting of these TagBIT-proteins with optimized TagBIT system were shown below. **(C-D)** TagBIT-based abundance quantification of the TagBIT-BZR1 (C) and TagBIT-Nramp5 (D). **(E-F)** Quantification of transcript level of each gene using qPCR. *ACTIN1* gene (Os03g0718100) was used as an internal control (the same below). For (D-F), values indicate means ± SD (n = 3). Statistical analyses were performed using Student's *t*-test. ** indicate $P<0.01$.

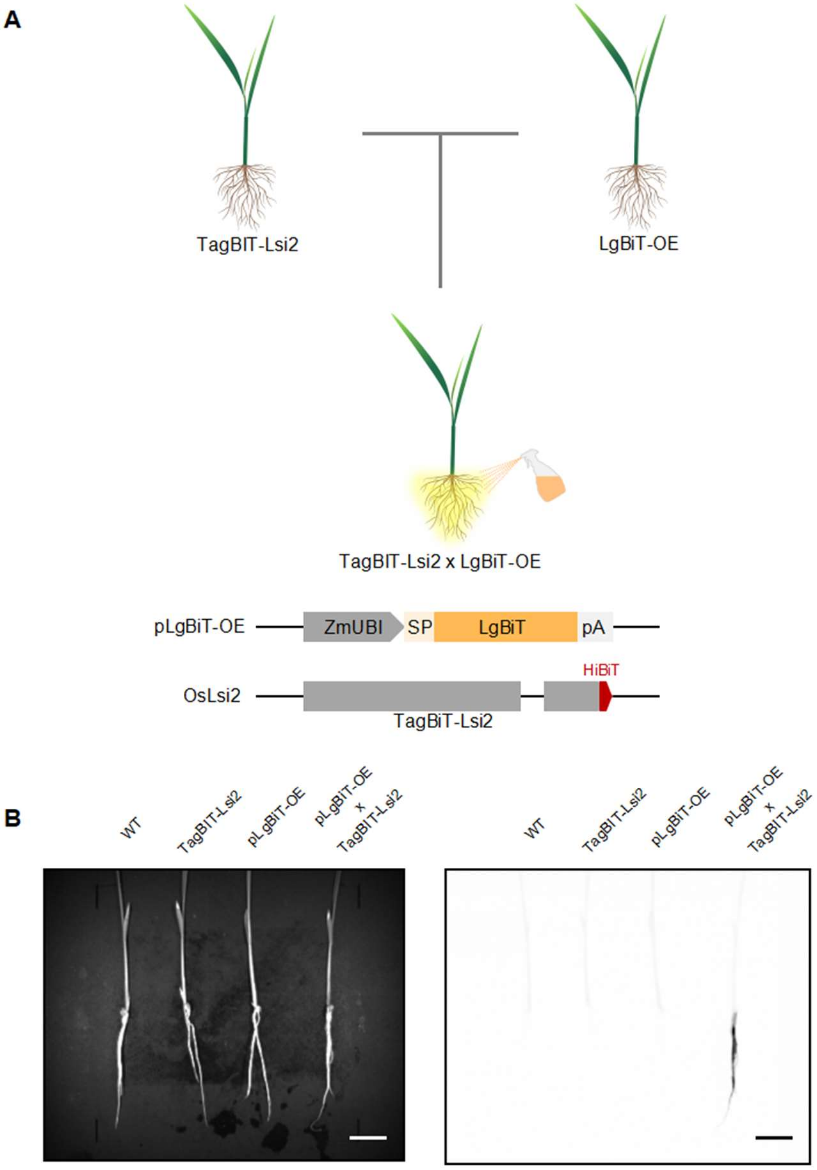

Supplementary Figure 5. Schematics to show TagBIT-based in vivo bioluminescence imaging.

(A) Procedure of TagBIT-based bioluminescence in plants. **(B)** Brightfield (left panel) and bioluminescence (right panel) images of selected plants.

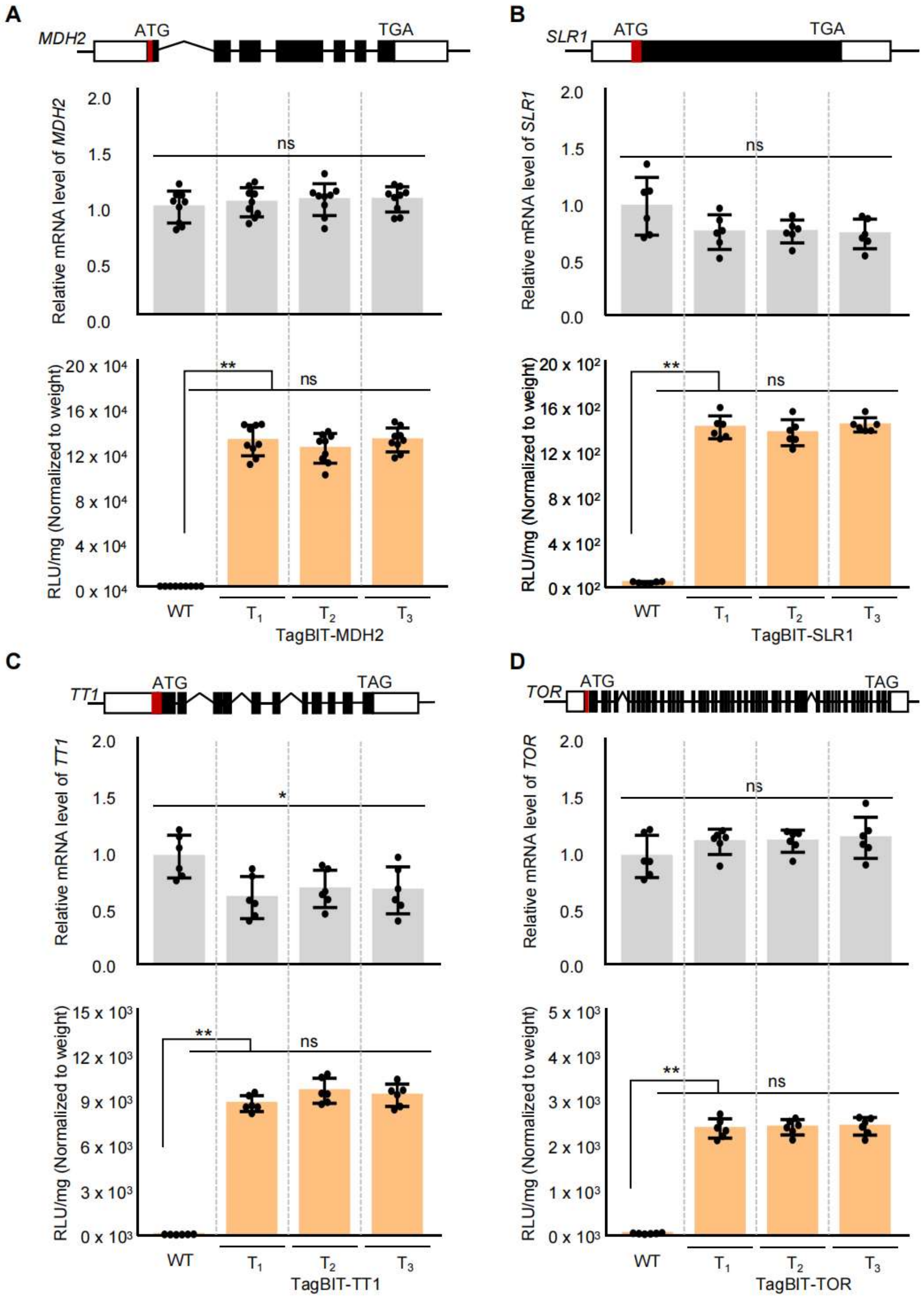

Supplementary Figure 6. Quantification of mRNA and protein level of representative TagBIT plants. **(A-D)** Comparison of the expression level of *MDH2*(A), *SLR1*(B), *TT1*(C) and *TOR*(D) between WT and TagBIT T1 to T3 lines. The rice *ACTIN* gene (Os03g0718100) was used as internal control. TagBIT-based abundance quantification of the TagBIT-proteins were shown below. HiBiT luminescence signal units were

stable across $T_1/T_2/T_3$ generations. Values indicate means $\pm$ SD (n = 9 or 6), each dot represents a single plant, *$P < 0.05$; **$P < 0.01$ (Student's *t*-test).

Supplementary table 1. Summary of TagBIT efficiencies in T_0 plants.

Gene	Insertion region	Methods	Editing efficiency	Knock-in efficiency[1]	Tagging efficiency[2]	Designed targets[3]	Description
MDH2	N-terminus	NHEJ	85/105 (80.9%)	22/105 (21%)	3/105 (2.9%)	CCGCTCCAATGGCGAAGGAACCG	Malate dehydrogenase 2
SLR1		NHEJ	36/46 (78.3%)	6/46 (13%)	4/46 (8.7%)	ATTACTAGCTATAGCTAGGTAGG	DELLA protein
TT1		NHEJ	58/71 (81.7%)	21/71 (29.6%)	6/71 (8.5%)	CCGGCGATGGGCGACAGCCAGTA	Proteasome α2 subunit
TOR		NHEJ	11/31 (35.5%)	5/31 (16.1%)	3/31 (9.7%)	CCGTCGCCATGAAGCCCTCGCCG	Target of Rapamycin
BZR1	C-terminus	NHEJ	39/59 (66.1%)	17/59 (28.8%)	11/59 (18.6%)	TCGGCGTCGGCGCGAAATGACGG	Brassinazole-resistant 1
Lsi2		NHEJ	24/51 (47.1%)	11/51 (21.6%)	2/51 (3.9%)	CCCCTCATCGGCAAGATCTGATC	Silicon efflux transporter
Nramp5		NHEJ	31/57 (54.4%)	10/57 (17.5%)	3/57 (5.3%)	CCAAGGTAGAGAAGAAGAAGATC	Natural Resistance-Associated Macrophage Protein 5

Note: [1] Knock-in efficiencies were determined by PCR; [2] Tagging efficiencies were determined by subcloning and sequencing; [3] The PAMs were marked in bold and the start codons (ATG) or stop codons (TAG/TGA) were underlined.

Supplementary table 2. Transmission frequencies of selected TagBIT lines.

Gene	Insertion region	Parental line			Progeny			Copy Number[2]
		Lines	Generation	Genotype	Total	Targeted[1]	Transmission	
MDH2	N-terminus	#2	T_0	Heterozygous/ Chimeric	48	13	27.1%	4
		#6	T_0	Heterozygous/ Chimeric	48	15	31.3%	3
		#2-37	T_1	Homozygous	24	24	100%	2
		#2-37-1	T_2	Homozygous	24	24	100%	1
TT1	N-terminus	#5	T_0	Heterozygous/ Chimeric	24	10	41.7%	2
		#19	T_0	Heterozygous/ Chimeric	24	6	25%	6
		#33	T_0	Homozygous	24	24	100%	5
		#5-1	T_1	Homozygous	24	24	100%	3
		#5-1-1	T_1	Homozygous	24	24	100%	1
TOR	N-terminus	#17	T_0	Heterozygous/ Chimeric	24	12	50%	4
		#17-1	T_1	Homozygous	24	24	100%	5
		#17-8	T_1	Homozygous	24	24	100%	3
		#17-8-1	T_2	Homozygous	24	24	100%	1
		#17-8-4	T_2	Homozygous	24	24	100%	1
SLR1	N-terminus	#19	T_0	Heterozygous/ Chimeric	24	10	41.7%	3
		#19-14	T_1	Heterozygous/ Chimeric	24	11	45.8%	2
		#19-23	T_1	Homozygous	24	24	100%	2
		#19-14-1	T_2	Homozygous	24	24	100%	1
BZR1	C-terminus	#7	T_0	Heterozygous/ Chimeric	24	7	29.2%	3
		#35	T_0	Heterozygous/ Chimeric	24	4	16.7%	3
		#7-5	T_1	Homozygous	24	24	100%	1
		#7-5-1	T_2	Homozygous	24	24	100%	1
Lsi2	C-terminus	#15	T_0	Heterozygous/ Chimeric	24	8	33.3%	4
		#44	T_0	Heterozygous/ Chimeric	24	11	45.8%	4
		#15-3	T_1	Homozygous	24	24	100%	1
Nramp5	C-terminus	#10	T_0	Heterozygous/ Chimeric	24	5	20.8%	4
		#10-3	T_1	Heterozygous/ Chimeric	24	9	37.5%	2
		#10-7	T_1	Homozygous	24	24	100%	2
		#10-8	T_1	Homozygous	24	24	100%	1
		#10-7-1	T_2	Homozygous	24	24	100%	1

Note: [1] The HiBiT-tagged lines were determined by in-vivo lysis assay. [2] The copy number was detected with qPCR, and the single copy gene *SPS1* (Os01g0919400) was used as an internal control.

Supplementary table 3. The primers used in this study.

Primer	Sequence (5'-3')	Experiments
MDH2-KIF	CGCTTCCAGAACCTTCTCG	Primers for Sanger sequencing
MDH2-KIR	GGCAAACCAAGAGAACGAGG	
TT1-KIF	CCTCACAAGCAACAACGAACG	
TT1-KIR	GCGGAACGGAAGCCAC	
TOR-KIF	TCCCTCTATCTCCGCCTCG	
TOR-KIR	AGCGGAATCCGTGGGCGA	
SLR1-KIF	CCTTCTCTTCTTCCCCTTCTT	
SLR1-KIR	CCTTGCACGACCCCATATC	
BZR1-KIF	GCGCGGAGTTCGAGTTC	
BZR1-KIR	TTGACCAGTACTGATCTAACAACA	
Lsi2-KIF	GCGGCGAACCTGATAGT	
Lsi2-KIR	GACGACGACGACGACGAG	
Nramp5-KIF	CGTCACCTTCGTCGCCGACTC	
Nramp5-KIR	GCACGTGGCATCCATAGAGG	
TOR-R	ATTCGACCAATTTAGAGCCC	
HiBiT-F	GCTGGCGGCTGTTCAAGAAGA	
MDH2-qF	GACTCCCAGTGGAGAGAAGC	Primers for qPCR detection
MDH2-qR	CTGCTGGACGGTAGAGATGA	
TOR-qF	ATGTGATGCAAGTACTTCGAAC	
TOR-qR	TTTGTAACTTGAGGGACTTCATTGA	
SLR1-qF	GCTCCAATGCCTACAAACA	
SLR1-qR	TTCTCCTCCACCCGGTAG	
BZR1-qF	CGGGAACTACAACCTCCCCAAGCA	
BZR1-qR	TCAGCAGCTGCGTTGACGAGC	
Actin1-qF	AAGACTGTAATACCTATTG	
Actin1-qR	ATAGAATAATCGCAACTC	
TT1-qF	CAATCTGGTGGTGTAAGACC	
TT1-qR	TCCAGGAGAAGTATGACC	
Nramp5-qF	CAGCAGCAGTAAGAGCAAGATG	
Nramp5-qR	GTGCTCAGGAAGTACATGTTGAT	
Lsi2-qF	ATCACCTTCCCCAAGTTCCT	
Lsi2-qR	CAGCTCCCTCCAGTACATGC	

Supplementary table 3. Continued

Primer	Sequence (5'-3')	Experiments
PT-uni-UP	p-G*G*AGGCAGCATGGTAAGCGGCTGGCGGCTGTTCAAGAAG ATTAGCGGATCCTCGG*G*C	Chemically modified dsODN
PT-uni-LW	p-G*C*CCGAGGATCCGCTAATCTTCTTGAACAGCCGCCAGCCG CTTACCATGCTGCCT*C*C	
PT-tor-UP	p-G*G*AGCCACCATGGTAAGCGGCTGGCGGCTGTTCAAGAAGA TTAGCGGATCCTC*G*G	
PT-tor-LW	p-C*C*GAGGATCCGCTAATCTTCTTGAACAGCCGCCAGCCGCT TACCATGGTGGCT*C*C	
Cas9-F	GACTGGGACCCTAAGAAGTACG	Detecting the T-DNA
Cas9-R	GGTAAACAGGTGGATGATATTCTC	
LgBiT-F	GGTGTTACTTCTGCAGATGGCTCCCTGCCTCCTCCTC	Primers for vector constructions
LgBiT-R	TCTATCGATCAATCAGAGACCGGTACCTCATGAATTTATGGTGA	
MDH2-F2	TCTCTCGCAAAACTCACCCAAAAGA	Primers for mutation detection
MDH2-R2	GAAAACCAGATCAAGCGACCATAA	
TT1-F2	CCCCTCACAAGCAACAACGAA	
TT1-R2	GACGAACCAGCGCACACGCTC	
TOR-F2	GCAAGCCAAGCCAGGTCG	
TOR-R2	AGCCTAAACTCAGCACGG	
SLR1-F2	TGCCTTCCTCTCTGATCACCTG	
SLR1-R2	AGAGGTCCGAGGGGTTGTAG	
BZR1-F2	CCCCTTCTTCGCGGTCT	
BZR1-R2	ATGAACAGTGAAGCCGTGAATA	
Lsi2-F2	CATGCATGCAGTGCTTCTTATGG	
Lsi2-R2	GCTGAGCTGATCTGATCACCA	
Nramp-F2	GCATGCTCATCAGTCGATACC	
Nramp-R2	GGACGTTGGCTCTGCCCTG	

Supplementary sequence 1. DNA sequence of pOE-LgBiT vector.

ZmUBI promoter, Signal peptide, pco-LgBiT, and NOS terminator sequences are shown below.

TGCAGCGTGACCCGGTCGTGCCCCTCTCTAGAGATAATGAGCATTGCATGTCTAAGTTATAAAAAATTA
CCACATATTTTTTTTGTCACACTTGTTTGAAGTGCAGTTTATCTATCTTTATACATATATTTAAACTTTACT
CTACGAATAATATAATCTATAGTACTACAATAATATCAGTGTTTTAGAGAATCATATAAATGAACAGTTAG
ACATGGTCTAAAGGACAATTGAGTATTTTGACAACAGGACTCTACAGTTTTATCTTTTTAGTGTGCATGT
GTTCTCCTTTTTTTTTGCAAATAGCTTCACCTATATAATACTTCATCCATTTTATTAGTACATCCATTTAGG
GTTTAGGGTTAATGGTTTTTATAGACTAATTTTTTTAGTACATCTATTTTATTCTATTTTAGCCTCTAAATTA
AGAAAACTAAAACTCTATTTTAGTTTTTTTATTTAATAATTTAGATATAAAATAGAATAAAATAAAGTGACT
AAAAATTAAACAAATACCCTTTAAGAAATTAAAAAAACTAAGGAAACATTTTTCTTGTTTCGAGTAGATAA
TGCCAGCCTGTTAAACGCCGTCGACGAGTCTAACGGACACCAACCAGCGAACCAGCAGCGTCGCGTC
GGGCCAAGCGAAGCAGACGGCACGGCATCTCTGTCGCTGCCTCTGGACCCCTCTCGAGAGTTCCGCT
CCACCGTTGGACTTGCTCCGCTGTCGGCATCCAGAAATTGCGTGGCGGAGCGGCAGACGTGAGCCGG
CACGGCAGGCGGCCTCCTCCTCCTCTCACGGCACGGCAGCTACGGGGGATTCCTTTCCCACCGCTCC
TTCGCTTTCCCTTCCTCGCCCGCCGTAATAAATAGACACCCCCTCCACACCCTCTTTCCCCAACCTCGT
GTTGTTCGGAGCGCACACACACACAACCAGATCTCCCCCAAATCCACCCGTCGGCACCTCCGCTTCAA
GGTACGCCGCTCGTCCTCCCCCCCCCCCCCTCTCTACCTTCTCTAGATCGGCGTTCCGGTCCATGGTT
AGGGCCCGGTAGTTCTACTTCTGTTCATGTTTGTGTTAGATCCGTGTTTGTGTTAGATCCGTGCTGCTA
GCGTTCGTACACGGATGCGACCTGTACGTCAGACACGTTCTGATTGCTAACTTGCCAGTGTTTCTCTTT
GGGGAATCCTGGGATGGCTCTAGCCGTTCCGCAGACGGGATCGATTTCATGATTTTTTTTGTTTCGTTG
CATAGGGTTTGGTTTGCCCTTTTCCTTTATTTCAATATATGCCGTGCACTTGTTTGTCGGGTCATCTTTTC
ATGCTTTTTTTTGTCTTGGTTGTGATGATGTGGTCTGGTTGGGCGGTCGTTCTAGATCGGAGTAGAATTC
TGTTTCAAACTACCTGGTGGATTTATTAATTTTGGATCTGTATGTGTGTGCCATACATATTCATAGTTACG
AATTGAAGATGATGGATGGAAATATCGATCTAGGATAGGTATACATGTTGATGCGGGTTTTACTGATGC
ATATACAGAGATGCTTTTTGTTCGCTTGGTTGTGATGATGTGGTGTGGTTGGGCGGTCGTTCATTCGTT
CTAGATCGGAGTAGAATACTGTTTCAAACTACCTGGTGTATTTATTAATTTTGGAACTGTATGTGTGTGT
CATACATCTTCATAGTTACGAGTTTAAGATGGATGGAAATATCGATCTAGGATAGGTATACATGTTGATG
TGGGTTTTACTGATGCATATACATGATGGCATATGCAGCATCTATTCATATGCTCTAACCTTGAGTACCT
ATCTATTATAATAAACAAGTATGTTTTATAATTATTTTGATCTTGATATACTTGGATGATGGCATATGCAGC
AGCTATATGTGGATTTTTTTAGCCCTGCCTTCATACGCTATTTATTTGCTTGGTACTGTTTCTTTTGTCGA
TGCTCACCCTGTTGTTTGGTGTTACTTCTGCAGATGGCTCCCTGCCTCCTCCTCGTCCTCTTCCTCCTC
CCGGCGCTCGCCACCGGCATGGTGTTCACCCTGGAGGACTTCGTCGGCGACTGGGAGCAGACGGCG
GCGTACAACCTCGACCAAGTGCTAGAACAGGGCGGCGTCTCGTCTTTGCTGCAAAATCTCGCCGTGTC
CGTTACGCCGATCCAGAGGATAGTCCGTAGTGGAGAAAATGCTTTAAAAATTGACATACATGTTATCATT
CCGTATGAGGGGCTTTCCGCAGATCAGATGGCTCAAATTGAGGAGGTATTTAAAGTGGTGTACCCTGTT
GATGATCACCACTTCAAGGTGATCCTTCCTTACGGCACACTTGTCATCGACGGGGTAACTCCCAACATG
CTGAACTACTTTGGTAGACCATATGAAGGCATCGCCGTTTTTGATGGGAAGAAGATCACCGTTACTGGA
ACATTGTGGAATGGTAACAAGATTATTGATGAGCGGCTCATTACACCAGATGGAAGCATGCTCTTCCGC
GTCACCATAAATTCATGAGGTACCGGTCTCTGATTGATCGATAGAGCTCGAATTTCCCCGATCGTTCAA
ACATTTGGCAATAAAGTTTCTTAAGATTGAATCCTGTTGCCGGTCTTGCGATGATTATCATATAATTTCTG
TTGAATTACGTTAAGCATGTAATAATTAACATGTAATGCATGACGTTATTTATGAGATGGGTTTTTATGAT

TAGAGTCCCGCAATTATACATTTAATACGCGATAGAAAACAAAATATAGCGCGCAAACTAGGATAAATTATCGCGCGCGGTGTCATCTATGTTACTAGATCGG

Supplementary file 1. Unprocessed image of DNA electrophoresis.

OsTT1 (TT1-KIF+TT1-KIR, 3% agarose)

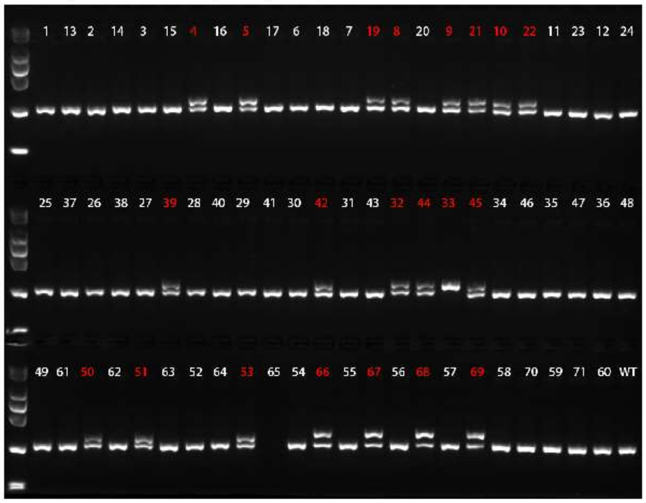

OsTOR (TOR-KIF+TOR-KIR, 3% agarose)

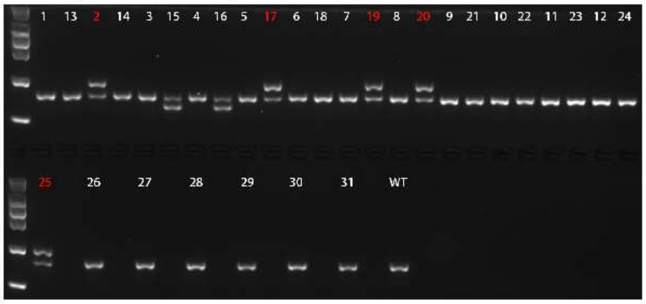

SLR1 (SLR1-KIF+SLR1-KIR, 3% agarose)

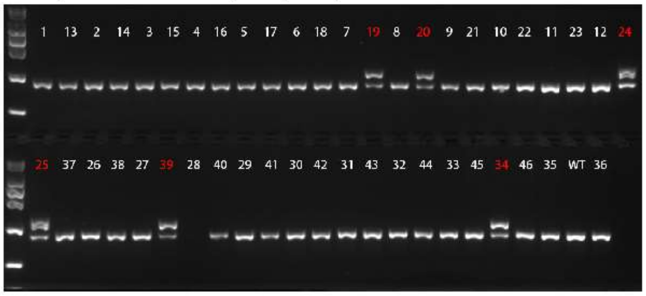

OsBZR1 (BZR1-KIF+BZR1-KIR, 3% agarose)

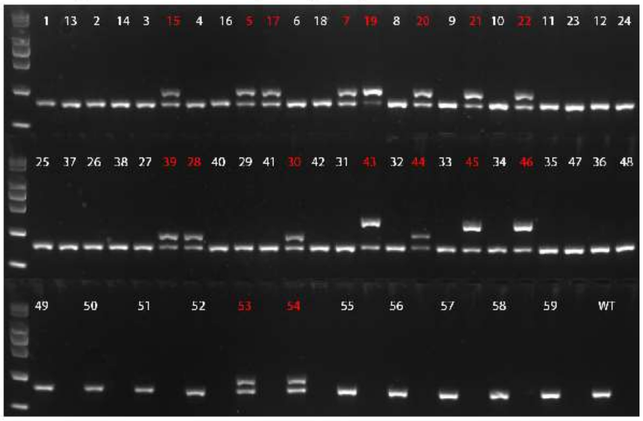

OsLsi2 (Lsi2-KIF+Lsi2-KIR, 3% agarose)

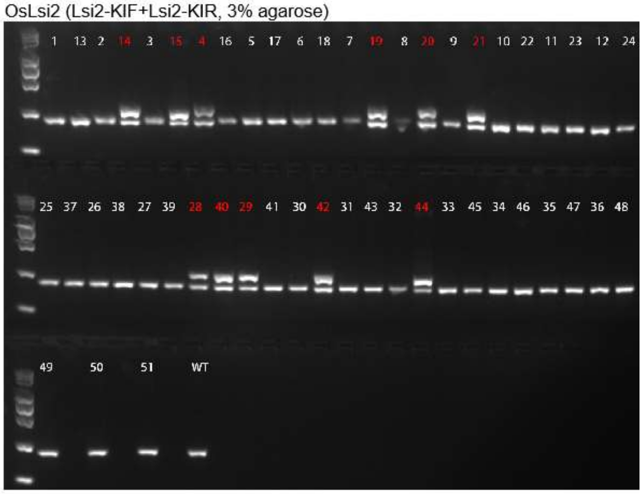

OsNramp5 (Nramp5-KIF+Nramp5-KIR, 3% agarose)

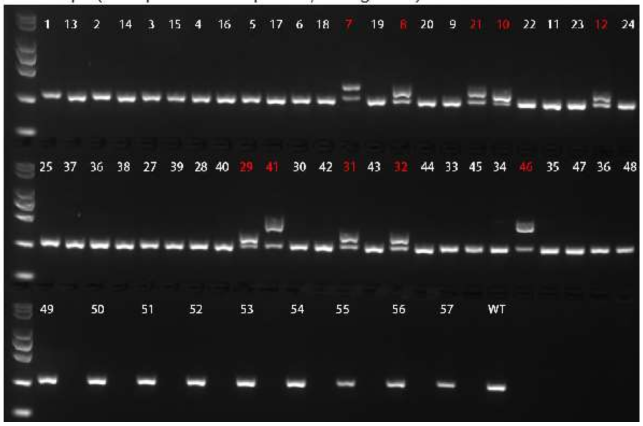

Note: The T_0 plantlets were detected with primer pairs (KIF+KIR), and the PCR products were investigated by 3% agarose gel electrophoresis. The plantlet harboring a larger amplicon was determined as a targeted knock-in plant.

Supplementary file 2. Unprocessed image of TagBIT blotting.

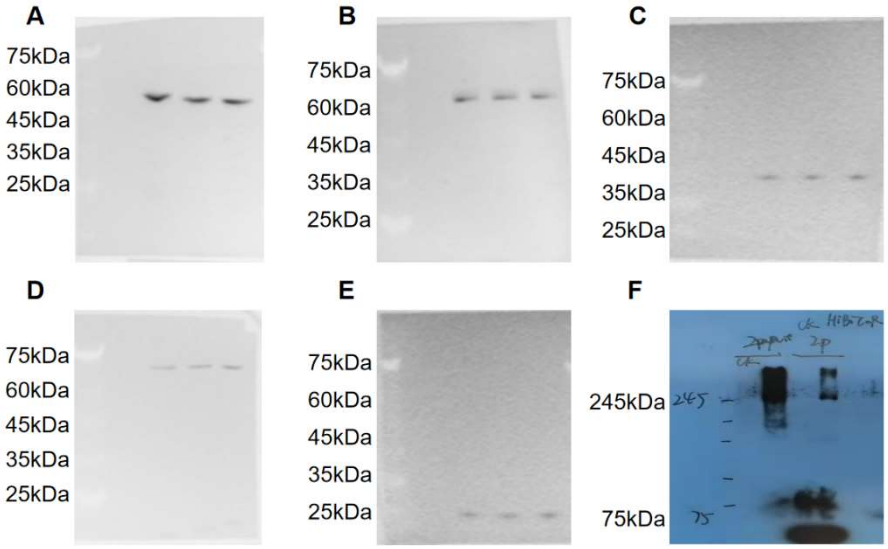

Note: (A-F) TagBIT-based protein blotting detection of the Lsi2 (A), Nramp5 (B), BZR1 (C), SLR1 (D), TT1 (E), and TOR (F) TagBIT lines.
